# Is population screening for abdominal aortic aneurysm cost-effective?

**DOI:** 10.1186/1471-2261-8-32

**Published:** 2008-11-18

**Authors:** Lars Ehlers, Jan Sørensen, Lotte Groth Jensen, Merete Bech, Mette Kjølby

**Affiliations:** 1 Institute of Public Health, Aarhus University, Denmark; 2 Centre for Applied Health Services Research and Technology Assessment, University of Southern Denmark, Denmark; 3 Centre for Public Health, Denmark

## Abstract

**Background:**

Ruptured abdominal aortic aneurysm (AAA) is responsible for 1–2% of all male deaths over the age of 65 years. Early detection of AAA and elective surgery can reduce the mortality risk associated with AAA. However, many patients will not be diagnosed with AAA and have therefore an increased death risk due to the untreated AAA. It has been suggested that population screening for AAA in elderly males is effective and cost-effective. The purpose of this study was to perform a systematic review of published cost-effectiveness analyses of screening elderly men for AAA.

**Methods:**

We performed a systematic search for economic evaluations in NHSEED, EconLit, Medline, Cochrane, Embase, Cinahl and two Scandinavian HTA data bases (DACEHTA and SBU). All identified studies were read in full and each study was systematically assessed according to international guidelines for critical assessment of economic evaluations in health care.

**Results:**

The search identified 16 cost-effectiveness studies. Most studies considered only short term cost consequences. The studies seemed to employ a number of "optimistic" assumptions in favour of AAA screening, and included only few sensitivity analyses that assessed less optimistic assumptions.

**Conclusion:**

Further analyses of cost-effectiveness of AAA screening are recommended.

## Background

The annual number of deaths attributable to ruptured abdominal aortic aneurysm (AAA) in Denmark is approximately 400–500 [1,2], corresponding to a mortality of 1–2% of males aged ≥ 65. Overall mortality from ruptured AAA is 80–90%; about half of patients die before they reach hospital [3-5]. Screening programmes that establish diagnosis through ultrasonography and offer elective AAA repair have been advocated because most patients die from undiagnosed ruptured AAA.

Four randomized controlled trials (RCTs) have shown a reduction in AAA-related mortality from screening programs aimed at elderly males [6]. An expected reduction in mortality is not sufficient for a screening program. The program should be acceptable to patients and, if publicly funded, should be "good value for money". That is, the decision to introduce such screening programs should be based on health effectiveness (good health outcomes) and cost-effectiveness (good value for money) [7].

The purpose of this study was to review cost-effectiveness studies of screening programs for AAA in elderly males, and to assess the evidence of cost-effectiveness of such programs.

## Methods

### Literature review

Systematic searches were undertaken in NHSEED, EconLit, Medline, Cochrane, Embase, Cinahl, and two Scandinavian health technology assessment databases (DACEHTA and SBU). The search was from 01.01.1997 to 01.06.2008. Search strategies (in Medline, Cinhahl, Cochrane and Embase) were thesaurus-guided: aortic-aneurysm-abdominal (MeSH) AND mass-screening (MeSH), and aortic-aneurysm-abdominal, added subheading "prevention and control". Free text search on AAA screening was also done. Reference lists of identified studies and other recent relevant publications were inspected for additional references.

### Inclusion criteria

Only studies in English or Scandinavian languages in peer-reviewed journals were included. Studies had to be a full health economic evaluation considering cost and effects (i.e. analyses of cost-effectiveness, cost-utility, and cost-benefit) of screening males for AAA.

### Analyses

Studies [8-23] were read in full. An assessment was made according to international guidelines for critical assessment of economic evaluations in health care [7] (Table 1). Special attention was given to included costs, and an overview of relevant cost headings were developed based on the reviewed articles (Table 2).

**Table 1:** Studies of cost-effectiveness of AAA screening

Nr.	Study	Alternative programs	ICER*	Comments
1	MASS 2002 (UK) [8]	Screening of men aged 65–74 years vs. no systematic screening strategy	GBP 28 400 per gained life-year or GBP 36 000 per QALY Price level: year 2000	Costs outside the health care sector not included. Different discount rates used for costs and effects. QOL after elective surgery assumed to be similar to normal population.
2	Kim et al. 2008 (UK) [9]	Screening of men aged 65–74 years vs. no systematic screening strategy	USD 19 500 per gained life-year Price level: year 2004–05	Only short-term hospital costs included. QOL after elective surgery assumed to be similar to normal population.
3	Lindholt et al. 2006 (DK) [10]	Screening of males aged 65–73 years vs. no systematic screening strategy	GBP 6 090 per gained life-year or GBP 10 793 per saved life Price level: year 2004	Only short-term hospital costs included. Costs and health effects not discounted.
4	Wilmink et al. 2003 (UK) [11]	Screening of males aged >50 years vs. no systematic screening strategy	USD 1 173 per gained life-year Price level: year 1995	Only short-term hospital costs included. Costs and health effects not discounted.
5	Hobbs et al. 2004 (UK) [12]	Screening of males aged >50 years vs. no systematic screening strategy	GBP 375–655 per gained life-year Price level: year?	Only short-term hospital costs included. Costs and health effects not discounted.
6	Ishikawa et al. 2004 (JP) [13]	Screening of males and females aged >60 years vs. no systematic screening strategy	USD 2 366 per detected AAA Price level: year?	Only short-term hospital costs included. Costs and health effects not discounted.
7	Lindholt et al. 2002 (DK) [14]	Screening of males aged 65–73 years vs. no systematic screening strategy	DKK 7 540 per gained life-year or DKK 67 855 per saved life Price level: year 1998	Only short-term hospital costs included. Costs and health effects not discounted. Long-term survival after elective surgery is assumed to be similar to normal population. Data for males aged 65–73 years are used as estimates for 65-year-old males.
8	Soisalon et al. 2001 (FI) [15]	Screening of first-degree relatives vs. no systematic screening strategy	USD 6 200 per gained life-year Price level: year 1987–89	Targeted screening of familial AAA. Only hospital costs included.
9	Kim et al. 2007 (UK) [16]	Screening of males aged 65 years vs. no systematic screening strategy	GBP 510 per gained life-year or GBP 676 per QALY Price level: year 2000	Only short-term hospital costs included. Long-term survival and QOL after elective surgery is assumed to be similar to normal population. Data for males aged 65–74 years used as estimates for 65-year-old males.
10	Wanhainen et al. 2005 (SE) [17]	Screening of males at age 60, 65 or 70 years vs. no systematic screening strategy	USD 10 474 per gained life-year or USD 13 900 per QALY (results for +65 year old) Price level: year 2003	Only short-term hospital costs and costs of lost time and travelling due to screening included. QOL after elective surgery assumed to be similar to normal population. Data for males aged 65–74 years (pooled estimates from heterogeneous studies) used as estimates for 65-year-old males.
11	Henrikson et al. 2005 (SE) [18]	Screening of men aged 65 years vs. no systematic screening strategy	EUR 7 760 per gained life-year or EUR 9,700 per QALY Price level: year 2003	QOL after elective surgery assumed to be similar to normal population. Data for males aged 65–74 years (pooled estimates from heterogeneous studies) used as estimates for 65-year-old males.
12	Boll et al. 2003 (NL) [19]	Screening of males aged 60–65 years vs. no systematic screening strategy	EUR 1 176 per gained life-years Price level: year 1997	Only short-term hospital costs included. Long-term survival after elective surgery assumed to be similar to normal population.
13	Lee et al. 2002 (US) [20]	Screening of males aged 70 years vs. no systematic screening strategy	USD 11 215 per QALY Price level: year?	Only short-term hospital costs included. Long-term survival and QOL after elective surgery assumed to be similar to normal population.
14	Conelly et al. 2002 (CA) [21]	Screening of males and females aged >50 years vs. no systematic screening strategy	CAD 741 per QALY Price level: year?	Only short-term hospital costs included. Long-term survival and QOL after elective surgery assumed to be similar to normal population.
15	Montreuil et al. 2008 (CA) [22]	Screening of males aged 65 years vs. no systematic screening strategy	CAD 6 194 per QALY Price level: year 2005	Only short-term hospital costs included. QOL after elective surgery assumed to be similar to normal population. Data for males aged 65–74 years used as estimates for 65-year-old males.
16	Silverstein et al. 2005 (US) [23]	Screening of males at age 65 years vs. no systematic screening strategy	USD 19 720 per QALY Price level: year?	Only short-term hospital costs included. Long-term survival and QOL after elective surgery assumed to be similar to normal population. Data for males aged 65–74 years used as estimates for 65-year-old males.

* ICER is not comparable between studies because results are based on different assumptions and methods

**Table 2:** Main types of costs associated with AAA screening

1. Invitation to screening (and re-invitations for non-attenders).
Includes clerical staff time, postage and stationery, cost of obtaining patient details, office space and equipment, overheads.
2. Ultrasonograpgy (and re-scan and surveillance).
Includes clinic staff time, staff travel cost, disposables, annuitization of capital expenditures, maintenance and service contracts, office space/charge of locations.

3. Surgery (pre-assessments for suitability, elective aneurysm repairs (as well as emergency surgery for ruptures) hospitalization).
Includes theatre time, time spent in intensive care and general ward, drugs, blood products, non-pathological investigations, graft inserted, and overheads.

4. Hospital and community care (short and long term)
Includes readmissions, graft surveillance and secondary procedures after surgery, visits to general practitioner, outpatient attendances and patient pathways due to surgical complications (dialysis-dependent renal failure, stroke, myocardial infarction, and major amputation).

5. Patient and family resources.
Includes transportation expenditures, medicine and time cost.

6. Resources in other sectors.
Includes social services (e.g. home help and nursing homes).

## Results

### Type of economic evaluation

Sixteen studies were included (Table 1). An extended description of the studies included is available as a web appendix (see "Additional file 1"). Five were from Scandinavia, five from the UK, two from USA and Canada, one from the Netherlands and one from Japan. Eight studies were designed as cost-effectiveness studies using objective health outcomes; eight were cost-utility studies with health outcomes expressed in quality-adjusted life-years (QALYs). Six studies were conducted alongside trials (termed "piggyback" in Table 2): three RCTs, two cohort studies and one case-control study. Ten studies employed decision analytic models.

### Costs associated with AAA screening

Based on the reviewed studies, the main types of costs associated with population screening, surveillance and surgery for AAA have been compiled as shown in Table 2.

There were great variations between the studies in the type of costs. Most studies included only short-term costs, i.e. it was implicitly assumed that there is no difference in the long-term costs between the alternatives. Only three studies [8,15,18] included long-term costs. The MASS study [8] and Soisalon-Soininen et al. [15] included costs of hospital and community care in a follow-up period of 4 years and 17 years, respectively. This included costs of hospital readmissions, visits to the general practitioner, outpatient attendances, and variations in patient pathways due to surgical complications (e.g. dialysis-dependent renal failure, stroke, myocardial infarction, and major amputation). Henriksson et al. [18] included an estimate of the average additional annual health care cost after surgery for AAA for the remaining lifetime of patients.

All studies included the costs of invitation to screening, ultrasonography, and surgery. Only three of the 16 studies included private cost to patients (e.g. transportation, time) [14,17,18]. No study included costs of social services (e.g. home help, nursing homes).

Cost amount varied considerably between studies (data not shown). An example is the cost of ultrasonography in the MASS study [8], which was more than double the cost in the study by Lindholt et al. [10] (even if adjusting for different cost year). Another example was transportation cost (mobile screening team), which varied by more than fourfold. The cost of surgery also varied. Differences in cost estimates can be partly explained by the different organizational arrangements and different circumstances due to geography. All studies lacked detailed reporting of cost estimates, and offered only limited transparency in cost calculations.

### Organizational assumptions underpinning economic calculations

Ultrasonography is the "gold standard" for AAA screening, and has been used in all AAA trials and decision analytic models. All studies assumed that ultrasonography was done by a mobile team of hospital specialists. Surveillance of patients with small AAAs was assumed to be handled by the same team. There were differences with regard to the: (i) number and type of screening locations; (ii) average distances of travel for the team and patients; and (iii) role of the general practitioner. Organizational models in all studies were described only superficially, which restricted transferability.

### Economic evaluations alongside trials (short-term cost-effectiveness)

Six economic evaluations were conducted alongside trials using patient-level data in evaluation of the cost-effectiveness of AAA screening. Three studies [8-10] used patient-level data from a RCT with a time perspective of 4, 5 and 7 years. The MASS study [8] estimated the incremental cost-effectiveness ratio (ICER) to be GBP 28 400 per gained life-year, or approximately GBP 36 000 per QALY. The authors concluded that this result was at the margin of acceptability according to National Health Service thresholds, but they expected cost effectiveness to improve over time. Kim et al. [9] used data from MASS trial after 7 years of follow up to estimate ICER at USD19 000; however this study only included short term hospital costs. Lindholt et al. [10] estimated ICER to be GBP 6 090 per gained life-year without long-term cost and discounting. The three other studies that used patient-level data (from local cohort studies or case-control studies) estimated ICER to be in the same order of magnitude, and suggested that screening may be cost-effective [11-13].

### Economic evaluations using decision analytic modelling (long-term cost-effectiveness)

Ten studies used decision analytic modelling to estimate long-term cost-effectiveness of AAA screening. The general conclusion from these studies was that AAA screening seems to be cost-effective, but the ICER varies considerably and a direct comparison of results is not possible due to considerable differences in the analytical basis. Studies employed different methods (types of model, time frame, and perspective) and different assumptions (sources of evidence for effect and transition probabilities, and cost assumptions) for their analysis.

### Assumptions about screening effectiveness

Half of the modelling studies were based on evidence of the effectiveness of AAA screening from RCTs. All used evidence from screening a group of men (typically males aged 65–79 years) to estimate effectiveness of screening a single age group (typically 65-year-old males). Other modelling studies were based on effectiveness data from trials with lower levels of evidence.

In general, the main advantage of screening is assumed to be an increase in the number of patients diagnosed with AAA and offered elective AAA repair. All studies assumed a constant risk of rupture depending on AAA size. The gained life-years/QALYs arise because the total number of elective surgeries increases, and the need for emergency surgery of ruptured AAA is therefore assumed to decrease.

### Assumptions about long-term survival after elective surgery

Most of the studies used national mortality rates for the average population as proxy for long-term survival after elective surgery, but some studies made more realistic assumptions. Wanheinen et al. [17] assumed that the mortality in treated AAA patients were 2.05 times the rates in the age-matched normal population because of high comorbidity rates in AAA patients. Lee et al. [20] adjusted the annual mortality rates to take account of expected excess mortality in patients with dialysis-dependent renal failure, stroke, myocardial infarction or major amputation. Henriksson et al. [18] and Soisalon-Soininen et al. [15] used local mortality data for AAA patients for 5 years and 17 years, respectively, and carried out survival analysis using statistical methods (Weibull or actuarial) in their estimation of life-years gained.

### Assumptions about quality of life (QOL) and QALY after elective surgery

Seven of the decision analytic studies calculated ICER as the incremental cost per gained QALY. These studies implicitly assumed that postoperative QOL (and after recovery) was similar to the QOL of the age-matched general population. Only one study (Lee et al. [20]) included the reduced QOL experienced by patients with major surgical complications.

Only two studies carried out sensitivity analyses of QOL assumptions. Wanheinen et al. [17] assumed a short-term reduction in QOL due to anxiety experienced before untrasonography. They estimated a reduction of 5% in the first-year QALY, which was outweighed by uncertainty in the long term. Henriksson et al. [18] did a multiway sensitivity analysis in which QOL was allowed to vary stochastically according to a pre-specified statistical distribution, but it was not possible to distinguish the effect.

## Discussion

The purpose of this review was to carry out a critical assessment of cost-effectiveness studies of screening older males for AAA. Based on our review of 16 studies, it seems that most of the health economic analyses of screening for AAA employed "optimistic" assumptions about the cost effectiveness of AAA screening.

The MASS cost-effectiveness study at four years [8] has the highest quality, but this single study does not provide enough information to assess the cost-effectiveness of AAA screening. MASS did not collect information on QALY gains, and endovascular aortic aneurysm repair was not used in the trial. The time perspective in MASS was four years, and a modelling approach is needed to assess long-term cost consequences and perform detailed sensitivity analyses.

In all published decision analytic models of AAA screening hypothetical patients with an AAA ≥ 5.5 cm were assumed to face a constant probability of rupture (average for males aged 65–79 years) no matter how many years they have had a large AAA. In cohort simulations such a constant probability of rupture gives a wrong distribution of death over time and a mean age of males having emergency surgery for ruptured AAA that is much too low. For instance in Denmark the mean age of death from ruptured AAA is 76 years (range 65–92) for males aged ≥ 65. One way to "build memory" into a model is to implement time dependency, but none of the modelling studies seems to have done so. Accordingly, when underestimating the age of males dying of ruptured AAA in the non-screening group the calculated number of "gained life-years" due to screening and avoiding ruptures is too high.

Most of the health economic studies of AAA screening only included short term hospital costs. Major implications for society due to comorbidity and severe surgical complications (e.g. stroke or chronic renal failure) were not included because most studies did not consider cost after hospital discharge. Patient pathways after such events can be very costly [24]. Furthermore, screening might induce extra long term cost of treatment of those unfit for surgery [9].

Economic evaluations did not incorporate evidence that the lives of tobacco smokers are generally shorter than those of the general population, and that they have a higher demand for health services (i.e. higher social and health care costs) and a lower QOL in the remaining life-years [25-27] (>90% of patients with AAA have a history of smoking [3-5]).

There has been considerable interest in smoking cessation programmes during the last decade. Successes in reducing the number of smokers have been linked to potential savings in future health care costs [25-27]. Economic evaluations of AAA screening seem to have ignored the relationship between tobacco smoking and AAA incidence. The incidence may even fall to levels that render population screening ineffective in terms of lives saved, let alone cost.

There is a lower prevalence of large AAAs in males who have never smoked, so the potential benefit from screening non-smokers is small. The USA Preventive Services Task Force recommends AAA screening in male smokers only for this reason [28]. The possibility of screening only male smokers could probably increase cost-effectiveness, although some authors argue the benefit from targeted screening is marginal compared with population screening [17].

All cost-utility studies assumed that patients with AAA could return to a QOL comparable with the average population: there is only poor evidence for this assumption. None of the randomised trials of AAA screening have collected evidence about QOL before and after screening and elective surgery in the screening group compared to the average population (i.e. the non-screening group). Only studies of QOL with poorer designs have been published.

Furthermore, the clinical literature of QOL after elective repair that are being referred to seems to be in conflict with public health evidence that smokers experience a lower QOL in their remaining years of life compared to the average population. At least more sensitivity analyses should have been done to evaluate the possibility of lower QOL due to comorbidity and severe surgical complications such as chronic renal failure, major amputation or stroke.

Various other factors likely to reduce cost-effectiveness were ignored in the economic evaluations. In most cases, cost calculations were based on open repair and not on endovascular aneurysm repair. Despite lack of evidence of cost-effectiveness, this method of treating AAAs is being used increasingly in many countries. It may reduce early mortality more effectively, but it may substantially reduce the cost-effectiveness of screening [29].

The possibility is that *ad hoc *detection of AAA cases will gradually increase as imaging (mostly ultrasonography) becomes more widely utilized for other reasons. This may reduce the prevalent pool of undiagnosed AAAs and hence screening effectiveness [30].

Our findings are in accordance with those of Campbell et al. [31]; they reviewed cost-effectiveness studies of AAA screening published between 1989 and 2003. They found that "eight of the nine population screening models have incorporated at least two assumptions, which would artificially favour a screening programme". This review by Campbell et al. has not been updated; the search period ended in 2003, and they excluded studies conducted alongside trials, which is a major source of evidence for cost-effectiveness. Our review includes ten new cost-effectiveness analyses, and only four studies overlap. Our review gives the "whole" picture of cost-effectiveness of AAA screening, which have not been presented before.

The individual conclusions of cost effectiveness cannot be rejected on the basis of this systematic review, but we can seriously challenge the assumptions on which the studies of cost-effectiveness are based. Our review leaves little doubt that the reported cost-effectiveness ratios of AAA screening in most cases have been too low.

Our findings should have implications for future research recommendations in cardiovascular medicine and surgery. It appears that existing analyses have overrated the advantages of AAA screening in terms of the expected number of gained life-years/QALYs and cost of a screening programme. Different patient pathways after surgery are ignored, and sensitivity analyses of long-term QOL after surgery are not satisfactory.

We therefore strongly recommend that an "updated" cost-effectiveness analysis should be carried out based on more realistic assumptions.

## Conclusion

This review indicates that most of the existing health economic evaluations have employed a number of "optimistic" assumptions in favour of AAA screening, and included only few sensitivity analyses that assessed less optimistic assumptions. Further analyses of cost-effectiveness of AAA screening are recommended.

## Competing interests

The authors declare that they have no competing interests.

## Authors' contributions

LE carried out the main study and drafted the manuscript. LS, MB, LGJ and MK participated in the analyses and discussion of the results. LGJ also carried out the literature search. All authors approved the final manuscript.

## Pre-publication history

The pre-publication history for this paper can be accessed here:



## Supplementary Material

Additional file 1 **Studies of cost-effectiveness of AAA screening.** The additional file is an extended version of Table 1 Studies of cost-effectiveness of screening for AAA. The extended version includes further details on the methods used and the primary sources of data for outcome. Click here for file
