# Supplementary material for: Is population screening for abdominal aortic aneurysm cost-effective?
Source: BMC Cardiovasc Disord. 2008 Nov 18;8:32. doi: 10.1186/1471-2261-8-32 (PMC2607250; doi:10.1186/1471-2261-8-32)
Supplement: Additional file 1 — Studies of cost-effectiveness of AAA screening. The additional file is an extended version of Table 1 Studies of cost-effectiveness of screening for AAA. The extended version includes further details on the methods used and the primary sources of data for outcome. [file 1471-2261-8-32-S1.doc]

# Additional file 1

**Studies of cost-effectiveness of AAA screening**

| Nr. | Study | Alternative programs | Method | Primary source of data for outcome | ICER* | Comments |
| --- | --- | --- | --- | --- | --- | --- |
| 1 | MASS 2002 (UK) [8] | Screening of men aged 65-74 years vs. no systematic screening strategy | (CEA) Piggyback Perspective: Health Care Sector Time horizon: 4 years | RCT, MASS, England | GBP 28 400 per gained life-year or  GBP 36 000 per QALY Price level: year 2000 | Costs outside the health care sector not included. Different discount rates used for costs and effects. QOL after elective surgery assumed to be similar to normal population. |
| 2 | Kim et al. 2008 (UK) [9] | Screening of men aged 65-74 years vs. no systematic screening strategy | (CEA) Piggyback Perspective: Health Care Sector Time horizon: 7 years | RCT, MASS, England | USD 19 500 per gained life-year Price level: year 2004-05 | Only short-term hospital costs included. QOL after elective surgery assumed to be similar to normal population. |
| 3 | Lindholt et al. 2006 (DK) [10] | Screening of males aged 65-73 years vs. no systematic screening strategy | (CEA) Piggyback  Perspective: Health Care Sector  Time horizon: 5 years | RCT, Viborg, Denmark | GBP 6 090 per gained life-year or  GBP 10 793 per saved life  Price level: year 2004 | Only short-term hospital costs included.  Costs and health effects not discounted. |
| 4 | Wilmink et al. 2003 (UK)  [11] | Screening of males aged >50 years vs. no systematic screening strategy | (CEA) Piggyback  Perspective: Health Care Sector Time horizon: 10 years | Cohort study Huntingdon District Birmingham, England | USD 1 173 per gained life-year Price level: year 1995 | Only short-term hospital costs included.  Costs and health effects not discounted. |
| 5 | Hobbs et al. 2004 (UK)  [12] | Screening of males aged >50 years vs. no systematic screening strategy | (CEA) Piggyback Perspective: Health Care Sector Time horizon: 10 years | Cohort study Huntingdon District Birmingham, England | GBP 375–655 per gained life-year Price level: year? | Only short-term hospital costs included.  Costs and health effects not discounted. |
| 6 | Ishikawa et al.  2004 (JP) [13] | Screening of males and females aged >60 years vs. no systematic screening strategy | (CEA) Piggyback  Perspective: Health Care Sector Time horizon: 10 years | Case-control study Gumna, Japan | USD 2 366 per detected AAA  Price level: year? | Only short-term hospital costs included.  Costs and health effects not discounted. |
| 7 | Lindholt et al. 2002 (DK)  [14] | Screening of males aged 65-73 years vs. no systematic screening strategy | (CEA) Model  Perspective: Health Care Sector Time horizon: 13 years | RCT, Viborg, Denmark | DKK 7 540 per gained life-year or  DKK 67 855 per saved life  Price level: year 1998 | Only short-term hospital costs included.  Costs and health effects not discounted. Long-term survival after elective surgery is assumed to be similar to normal population. Data for males aged 65-73 years are used as estimates for 65-year-old males. |
| 8 | Soisalon et al. 2001 (FI) [15] | Screening of first-degree relatives vs. no systematic screening strategy | (CEA) Model Perspective: Health Care Sector Time horizon: 17 years | Case-control study Helsinki, Finland | USD 6 200 per gained life-year  Price level: year 1987–89 | Targeted screening of familial AAA.  Only hospital costs included. |
| 9 | Kim et al. 2007 (UK)  [16] | Screening of males aged 65 years vs. no systematic screening strategy | (CUA) Markov model  Perspective: Health Care Sector Time horizon: 30 years | RCT, MASS, England | GBP 510 per gained life-year or GBP 676 per QALY  Price level: year 2000 | Only short-term hospital costs included.  Long-term survival and QOL after elective surgery is assumed to be similar to normal population. Data for males aged 65-74 years used as estimates for 65-year-old males. |
| 10 | Wanhainen et al. 2005 (SE) [17] | Screening of males at age 60, 65 or 70 years vs. no systematic screening strategy | (CUA) Markov model  Perspective: Societal  Time horizon: 35 years | Systematic review and pooling of estimates | USD 10 474 per gained life-year or  USD 13 900 per QALY (results for +65 year old)  Price level: year 2003 | Only short-term hospital costs and costs of lost time and travelling due to screening included.  QOL after elective surgery assumed to be similar to normal population. Data for males aged 65–74 years (pooled estimates from heterogeneous studies) used as estimates for 65-year-old males. |
| 11 | Henrikson et al. 2005 (SE)  [18] | Screening of men aged 65 years vs. no systematic screening strategy | (CUA) Markov model Perspective: Societal Time horizon: 40 years | Systematic review and pooling of estimates | EUR 7 760 per gained life-year or EUR 9,700 per QALY Price level: year 2003 | QOL after elective surgery assumed to be similar to normal population. Data for males aged 65–74 years (pooled estimates from heterogeneous studies) used as estimates for 65-year-old males. |
| 12 | Boll et al.  2003 (NL) [19] | Screening of males aged 60–65 years vs. no systematic screening strategy | (CEA) Markov model Perspective: Health Care Sector Time horizon: "lifetime" | Review | EUR 1 176 per gained life-years Price level: year 1997 | Only short-term hospital costs included. Long-term survival after elective surgery assumed to be similar to normal population. |
| 13 | Lee et al. 2002 (US) [20] | Screening of males aged 70 years vs. no systematic screening strategy | (CUA) Markov model  Perspective: Health Care Sector Time horizon: "lifetime" | Review | USD 11 215 per QALY Price level: year? | Only short-term hospital costs included. Long-term survival and QOL after elective surgery assumed to be similar to normal population. |
| 14 | Conelly et al. 2002 (CA) [21] | Screening of males and females aged >50 years vs. no systematic screening strategy | (CUA) Model Perspective: Health Care Sector Time horizon: "lifetime" | Review | CAD 741 per QALY Price level: year? | Only short-term hospital costs included. Long-term survival and QOL after elective surgery assumed to be similar to normal population. |
| 15 | Montreuil et al. 2008 (CA) [22] | Screening of males aged 65 years vs. no systematic screening strategy | (CUA) Model Perspective: Health Care Sector Time horizon: "lifetime" | Review | CAD 6 194 per QALY Price level: year 2005 | Only short-term hospital costs included.  OL after elective surgery assumed to be similar to normal population. Data for males aged 65–74 years used as estimates for 65-year-old males. |
| 16 | Silverstein et al. 2005 (US) [23] | Screening of males at age 65 years vs. no systematic screening strategy | (CUA) Markov model Perspective: Health Care Sector Time horizon: 20 years | Systematic review | USD 19 720 per QALY  Price level: year? | Only short-term hospital costs included. Long-term survival and QOL after elective surgery assumed to be similar to normal population. Data for males aged 65–74 years used as estimates for 65-year-old males. |

* ICER is not comparable between studies because results are based on different assumptions and methods
